# Internet Search Limitations and Pandemic Influenza, Singapore

**DOI:** 10.3201/eid1610.100840

**Published:** 2010-10

**Authors:** Alex R. Cook, Mark I.C. Chen, Raymond Tzer Pin Lin

**Affiliations:** Author affiliations: National University of Singapore, Singapore (A.R. Cook, M.I.C. Chen, R.T.P. Lin);; Tan Tock Seng Hospital, Singapore (M.I.C. Chen);; Duke-NUS Graduate Medical School, Singapore (M.I.C. Chen);; Ministry of Health, Singapore (R.T.P. Lin)

**Keywords:** Internet, influenza, viruses, data mining, bioinformatics, pandemic (H1N1) 2009, surveillance, Singapore, letter

**To the Editor**: In the past few years, several publications have reported that Internet search queries may usefully supplement other, traditional surveillance programs for infectious diseases (*1**–**3*). The philanthropic arm of Google offers Flu Trends, a site that provides up-to-date estimates of influenza activity in 20 countries of the Pacific Rim and Europe (*4*) by using data mining techniques to find good predictors of historic influenza indicators (*1*).

This service has yet to be extended to other countries and other diseases because access to official surveillance data is required, among other reasons. However, another Google service, Insights for Search, enables users to find and download time-series data of relative counts of arbitrary searches for a large number of countries (*5*). Pelat et al. have shown that a few, well-chosen searches on Google Insights provide data that closely correlate with French surveillance data for seasonal influenza, chickenpox, and gastroenteritis (*3*). Although Internet searches appear to be a promising tool for public health surveillance, our experience from using Google Insights in the context of pandemic (H1N1) 2009 in Singapore suggests it has important limitations.

In Singapore, the recent pandemic caused an outbreak that peaked at the start of August 2009; the first confirmed importation was at the end of May and first confirmed unlinked case was at the end of June. However, the number Google searches for “influenza,” “H1N1,” “swine flu,” and similar terms (in English and Chinese), as well as symptoms associated with the disease, peaked much earlier than did the number of cases (Figure). The number of searches surged after newsworthy events but was low during the epidemic itself and had declined to about 20% of maximum search volume by the time of the actual peak, as shown by traditional surveillance. Furthermore, no discernible local maxima were observed that corresponded to the peak in case data. In contrast, alternative traditional measures of influenza incidence—prevalence of the novel strain among viral samples and general practice surveillance (*6**,**7*)—provide a consistent description of the outbreak.

**Figure:**
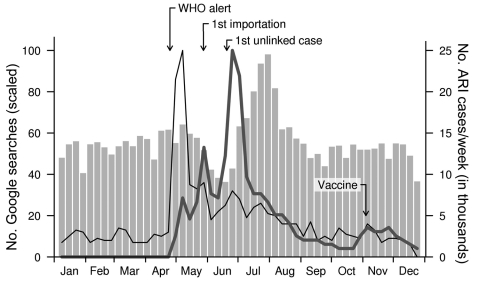
Number of Google searches conducted for “influenza” (black lines) and “H1N1” (gray lines) compared with number of acute respiratory infections (ARI, gray bars) reported in government clinics, Singapore, 2009. During the outbreak of pandemic (H1N1) 2009, Google search activity surged in response to newsworthy events (the World Health Organization [WHO] alert, first importation and unlinked local case, release of vaccine) but dropped substantially by the time most infections occurred in August. Other search patterns, such as for “swine flu” and simplified Chinese language terms for swine flu and influenza, were similarly disassociated with actual disease incidence.

This finding echoes a major point raised by Carneiro and Mylonakis (*2*), namely, that without adjusting for spikes driven by disease publicity rather than the disease itself, Internet searches may lose much of their value in supplementing traditional surveillance measures. Our experience is that using Google Insights to survey a disease may not work well for diseases with considerable media exposure, in particular, emerging diseases such as pandemic (H1N1) 2009 or severe acute respiratory syndrome. Such outbreaks may require the more sophisticated approach used by Flu Trends, should it be extended to other diseases and more corners of the globe. We agree with Pelat et al. (*3*) that Google Insights may work well for less-publicized infectious diseases. The dividing line between well-publicized and unpublicized diseases may, however, remain ambiguous. Thus, to ensure that web search data reflect disease incidence requires validation against traditional surveillance, although in that situation, the availability of corroborating traditional methods of surveillance limits the value of web-query data.
